# Effects of mind-body interventions on polycystic ovary syndrome: a comprehensive meta-analysis

**DOI:** 10.1186/s13048-024-01477-2

**Published:** 2024-07-25

**Authors:** Kun Zhao, Liuyan Nie, Xiangming Ye, Xiaoyan Hu

**Affiliations:** 1 Center for Rehabilitation Medicine, Rehabilitation & Sports Medicine Research Institute of Zhejiang Province, Department of Rehabilitation Medicine, Affiliated People’s Hospital, Zhejiang Provincial People’s Hospital, Hangzhou Medical College, 158 Shangtang Road, Hangzhou, Zhejiang 310014 China; 2 Department of Rheumatology, Sir Run Run Shaw Hospital, Zhejiang University School of Medicine, Hangzhou, China

**Keywords:** Mind-body interventions, Polycystic ovary syndrome, Endocrine and glycolipid metabolism, Meta-analysis, Randomized controlled trial

## Abstract

**Background:**

Mind-body interventions (MBI) have emerged as a potential therapeutic approach, but their effectiveness in the treatment of Polycystic Ovary Syndrome (PCOS) remains inconclusive. This study systematically evaluates the effectiveness of MBI on quality of life, anthropometry, androgen secretion, glucose, and lipid metabolism in PCOS.

**Methods:**

A computer search was conducted across three databases: PubMed, the Cochrane Library, and EMBASE, to identify randomized controlled trials (RCTs) related to MBI for PCOS from their inception until July 2024. DerSimonian and Laird’s random-effects model and Stata 17.0 software was employed for our meta-analysis.

**Results:**

Twelve RCTs were included. MBI significantly improved PCOSQ subscale scores, including emotional disturbances (MD: 7.75, 95% CI: 6.10 to 9.40), body hair (MD: 2.73, 95% CI: 0.54 to 4.91), menstrual problems (MD: 3.79, 95% CI: 2.89 to 4.69), and weight (MD: 1.48, 95% CI: 0.03 to 2.93). Furthermore, there was a reduction in depression levels (MD: -1.53, 95% CI: -2.93 to -0.13). Sensitivity analysis confirmed the robustness of PCOSQ-Emotional disturbances and PCOSQ-Menstrual problems, with a high GRADE level of evidence for these subscales. Secondary outcome measures, including waist-hip ratio, fasting blood glucose, and HOMA-IR exhibited statistically significant differences. Subgroup analysis revealed that obesity could influence treatment outcomes.

**Conclusion:**

MBI can serve as an alternative therapy, modulating effect on the quality of life and depression in PCOS patients. Future well-designed, high-quality, and large-scale studies should be conducted to thoroughly assess the impact of different Mind-Body Interventions (MBI) on various PCOS phenotypes.

**Trial registration:**

PROSPERO (CRD42023472035).

**Supplementary Information:**

The online version contains supplementary material available at 10.1186/s13048-024-01477-2.

## Background

Polycystic ovary syndrome (PCOS) is a gynecological condition that primarily affects women of childbearing age, often leading to infertility [1]. It is a complex set of clinical symptoms characterized by reduced ovulation, irregular menstrual cycles, biochemical hyperandrogenism (elevated circulating androgens, such as testosterone, or increased free androgens), clinical hyperandrogenemia (visible effects of androgens on body tissues, including hirsutism or excessive hair growth), and infertility [1]. In addition to hormonal imbalances, PCOS is closely associated with metabolic disorders, including dyslipidemia, insulin resistance, type II diabetes, and certain cardiovascular diseases [2, 3]. Some studies have demonstrated the interdependence and inseparability of insulin and androgens in PCOS; an elevation in one hormone can impact the abnormal secretion of the other, further complicating the pathogenesis of PCOS [4]. Furthermore, research has shown that individuals with PCOS are susceptible to abnormal lipid metabolism, resulting in increased levels of total cholesterol, triglycerides (TG), and low-density lipoprotein cholesterol (LDL), as well as reduced levels of high-density lipoprotein (HDL) [5, 6]. The altered endocrine metabolism in PCOS can lead to changes in body image, potentially causing adverse psychological and emotional effects [6, 7].

Currently, PCOS management primarily focuses on improving weight management and regular monitoring, including lifestyle interventions such as dietary and exercise modifications, pharmacological interventions (use of contraceptives, androgen-lowering medications, insulin resistance management, and ovulation-inducing drugs), and surgical interventions (e.g., laparoscopic ovarian drilling) [8–11]. However, the existing treatment strategy has certain limitations. For instance, lifestyle changes alone may not completely address reproduction or hormone levels [12]. Although medications and surgeries have some therapeutic effects, they carry various risks of adverse reactions, such as vascular thromboembolism, potential feminization of male offspring, severe gastrointestinal adverse events, ovarian hyperstimulation syndrome, anesthesia-related incidents, postoperative infections, and pelvic adhesions [13].

Hence, the exploration of alternative treatments is crucial. Recent studies have highlighted the potential benefits of mind-body interventions (MBI) for PCOS patients [14, 15]. MBI comprises various techniques, including meditation, relaxation, breathing techniques, tai chi, yoga, cognitive-behavioral therapy (CBT), qigong, hypnosis, biofeedback, and visual imagery [16]. It encompasses a comprehensive approach that leverages the mind to regulate bodily functions, promoting overall health by focusing on the intricate relationship between the brain, mind, body, and behavior [16]. Notably, MBI has been shown to improve weight loss and quality-of-life scores and to alleviate psychological anxiety [17]. This is achieved through low-intensity exercises that help regulate psychological emotions by reducing sympathetic tension, cortisol levels, and overall stress [18].

In prior research, the impact of yoga on PCOS had been summarized [19]; however, there was a limited number of randomized controlled trials, and the inclusion of pre-post clinical trials or case series in the synthesis raised the possibility of bias in the combined results [19]. Nevertheless, in recent years, with the publication of more randomized controlled trials, we now have access to a greater pool of reliable data for a more precise assessment of the effects of mind-body therapy on PCOS [20, 21].

In this study, we aim to comprehensively summarize the effects of MBI on various aspects of PCOS, including anthropometrics, endocrine parameters, glucose metabolism and blood lipid levels, quality of life, anxiety, depression, and stress levels. This meta-analysis seeks to provide valuable clinical evidence to inform the diagnosis and treatment of PCOS.

## Materials and methods

This systematic review is reported according to the updated Preferred Reporting Items for Systematic Reviews and Meta-analyses (PRISMA) statement for meta-analysis [22] and was conducted following an a priori-established protocol registered with PROSPERO (CRD42023472035).

### Search strategy

In this study, we conducted a comprehensive search in three electronic databases (EMBASE, PubMed, and Cochrane Library) up to July 6, 2024. We employed a combination of Medical Subject Headings (MeSH) or EMBASE Tree (EMTREE) terms along with text words. Additionally, we supplemented our search by consulting the references of included studies, as well as referring to previous meta-analyses or systematic reviews. For detailed search strategies, please refer to Table S1 in the Supplement.

### Inclusion and exclusion criteria

(1) All patients with PCOS met the diagnostic criteria as established by the National Institutes of Health [23], the Rotterdam criteria [24], or were clinically diagnosed by experienced physicians.

(2) The interventions primarily encompassed MBI, which were categorized as follows: meditation, relaxation, breathing techniques, tai chi, yoga, CBT, qigong, hypnosis, biofeedback, and visual imagery among other similar therapeutic approaches.

(3) The control group received one of the following interventions: no specific treatment (blank group), routine treatment or training, simple lifestyle modifications, completion of questionnaires, or standard medical advice.

(4) Outcome measures in this study were categorized into primary and secondary outcomes. Primary outcome measures consisted of PCOSQ (Polycystic Ovary Syndrome Questionnaire) subscale scores, encompassing emotional disturbances, body hair, infertility, menstrual problems, and weight [25]. Additionally, changes in depression, anxiety, and stress were assessed using any instruments and included as primary outcomes. Secondary outcome indicators covered included:

1. Anthropometrics measures: Weight, Body Mass Index (BMI), waist circumference, hip circumference, and waist-hip ratio.
2. Endocrine parameters: modified Ferriman-Gallwey (mFG)score, Follicle- Stimulating Hormone (FSH), Luteinizing Hormone (LH), LH/FSH ratio, Sex Hormone-Binding Globulin (SHBG), Dehydroepiandrosterone Sulfate (DHEAS), Total testosterone, and Free testosterone.
3. Markers related to glucose metabolism and blood lipid levels (Fasting insulin, Fasting blood glucose, Homeostatic Model Assessment for Insulin Resistance (HOMA-IR), triglycerides, HDL, LDL, and Total cholesterol).

(5) The study design was published as randomized controlled trial(RCT). The language in the literature was limited to English.

(6) Conference abstracts, and studies registered in clinical trial registries without available results or with missing valuable data that could not be transformed for analysis were excluded.

### Study selection and data extraction

Data selection was mainly carried out by two review authors (KZ and LYN). The titles and abstracts retrieved from the database and full-text articles were independently assessed and disagreements were resolved through consensus or referral to a third reviewer (XYH). The level of agreement between authors was determined using Cohen’s κ statistics. The main contents of each included literature data are: basic information of the article (author, country, and year of publication), participants (average age, and sample size), detailed information about MBI (type, dosage form, dose, and treatment time), comparison method and outcome parameters. If some research data are missing, we can contact the author by email or telephone to supplement the missing data.

### Quality assessment

In this study, two reviewers (KZ and LYN) assessed the quality of the included studies using the Risk of Bias 2 (RoB 2) tool, as recommended by Cochrane [26]. This tool evaluates the risk of bias in five domains: bias arising from the randomization process, bias due to deviations from intended interventions, bias due to missing outcome data, bias in the measurement of the outcome, and bias in the selection of the reported result. An overall risk of bias assessment was also conducted for each study. The Grading of Recommendations Assessment, Development and Evaluation (GRADE) approach was used to assess the certainty of evidence by evaluating the study strengths and limitations in different areas (risk of bias, publication bias, inconsistency, indirectness, and imprecision) [27]. GRADEpro GDT software was used to present the results.

### Statistical analysis

In this study, considering the existence of clinical heterogeneity (e.g., mode of intervention, participant characteristics), we employed the DerSimonian and Laird random-effects model for our meta-analysis [26]. Given that all outcome measures in our analysis are continuous variables, we initially transformed the data into mean and standard deviation (SD) format. Subsequently, for data synthesis, we employed mean differences (MD) with 95% Confidence Intervals (CI) for outcome measures that had consistent units. Standardized mean differences (SMD) were used for outcome measures with varying units to facilitate meaningful comparisons. The level of statistical significance was set at *P* < 0.05 and all statistical tests were two-sided. Statistical parameter I^2^ was used to examine the heterogeneity of the effect sizes and with values higher than 50% indicating substantial heterogeneity [26]. Subgroups were defined by three categorical moderators: the type of mind-body intervention, whether the participants were overweight or not, and the assessment of risk of bias. Sensitivity analysis was conducted by systematically excluding studies one by one. A funnel plot and Egger’s regression were used to examine the presence of publication bias, particularly when the number of included studies exceeded 10 for specific outcome [26]. The software used in this study is Stata 17.0 software (StataCorp., T.X., USA).

## Results

### Selection of literature

**Fig. 1:**
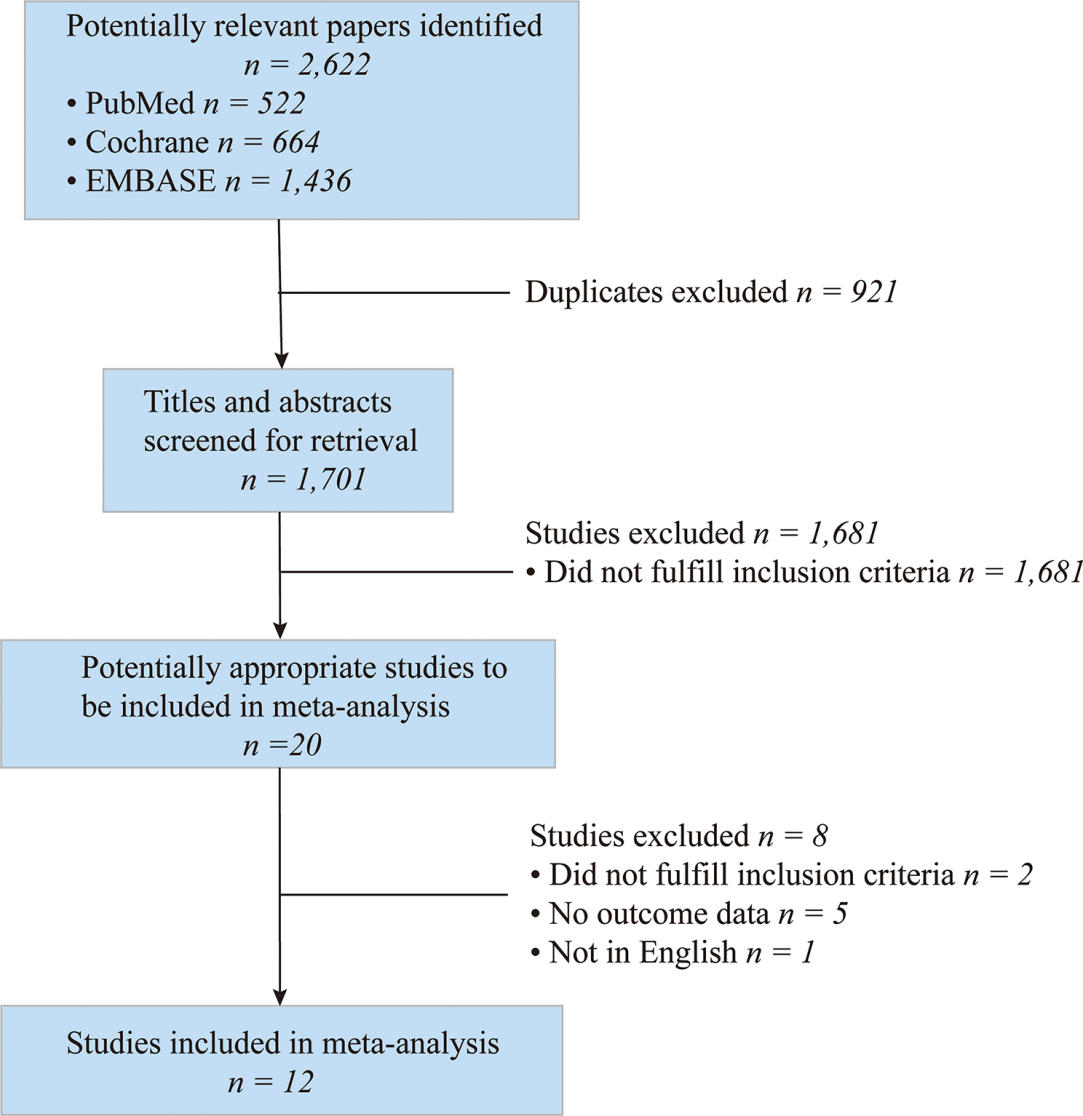
Flow diagram showing the process of literature selection

In this study, a total of 2,622 articles were initially identified, including PubMed (522), Cochrane Library (664), and EMBASE (1,436). Subsequently, based on the inclusion criteria and exclusion criteria (κ = 0.82), 2,602 studies were excluded first, while 921 were duplicate studies. After carefully reviewing the full text (κ = 0.97), it was determined that the following studies did not meet the inclusion criteria and were consequently excluded from the analysis: four studies with missing data [28–31]; one study was not available in English [32]; two studies did not meet the specified inclusion criteria [33, 34]; one study exhibited data anomalies, with the average score surpassing the total possible score of the PCOSQ subscale. Despite our efforts to contact the authors, we were unable to obtain a resolution or data correction method [35]. Therefore, 12 studies were finally included in this study [20, 21, 36–45] (Fig. 1).

### Study characteristics and quality

The characteristics of the included studies are presented in Table 1. The distribution of these studies by country was as follows: the United States [41, 43], China [20, 45], and Iran [21, 44] each contributed two studies, while India provided four articles pertaining to the same group of patients but with differing outcomes, which were consolidated into one study [36–39]. Additionally, Greece [40] and Denmark [42] each contributed one study. Four of these studies specifically focused on obese women with PCOS [20, 41–43], with the sample sizes of included studies ranging from 18 to 85 individuals. The interventions included three with yoga [36–39, 43, 44], two with cognitive-behavioral therapy [21, 41], and others like mindfulness stress management technology, motivational interviewing, tai chi, and Integrative Body-Mind-Spirit (I-BMS) intervention model.

**Table 1:** The characteristics of included studies

Study	Country	Diagnostic criteria	overweight women	Body mind therapy group			Control group			Outcome
				*N*	Age (year) mean ± sd	Type of treatment ( Number of sessions/weeks, Session duration(hours), Intervention duration (weeks))	*N*	Age (year)	Intervention	
Nidhi R, 2012, 2013	India	Rotterdam 2003 consensus criteria	No	42	16.22 ± 1.13	yoga (1, 7, 12)	43	16.22 ± 0.93	Physical Training	PCOSQ domains, Anxiety (STAI-State), BMI, Waist Circumference, Hip circumference, Waist-hip ratio, mFG score, FSH, LH, LH/FSH, Total testosterone Fasting insulin, Fasting blood glucose, HOMA-IR, triglycerides, HDL, LDL
Stefanaki C, 2015	Greece	Rotterdam 2003 consensus criteria	No	23	23.4 ± 4.62	mindfulness stress management techniques (7, 0.5, 8)	15	28.3 ± 7.20	questionnaire survey	Depression (DASS 21), Anxiety (DASS21), Stress (PSS-14), PCOSQ domains
Cooney LG, 2018	USA	National Institutes of Health criteria for PCOS	Yes	19	29.0 ± 5.93	cognitive-behavioral therapy (1, 0.5, 8)	12	32.0 ± 3.19	lifestyle modification	PCOSQ domains, Depression (CES-D), Anxiety (STAI-State), Stress (PSS), Weight, BMI, Waist circumference, Hip circumference, Waist-hip ratio, Total testosterone, Free testosterone, SHBG, HOMA-IR, triglycerides, HDL, LDL, Total cholesterol
Moeller LV, 2019	Denmark	Rotterdam 2003 consensus criteria	Yes	14	34 [23, 38] ^*^	motivational interviewing + standard advice (0.5, -, 24)	14	27 [22, 30] ^*^	standard advice	PCOSQ domains, Depression (MDI), Weight, BMI
Patel V, 2020	USA	Rotterdam 2003 consensus criteria	Yes	18	30.9 ± 1.2	yoga (1, 3, 12)	9	31.2 ± 2.3	control	Anxiety (BAI), Depression (BDI-II), BMI, Waist-hip ratio, mFG score Free testosterone, DHEAS, Fasting blood glucose, Fasting insulin, HOMA-IR
Mohseni M, 2021	Iran	Rotterdam 2003 consensus criteria	No	31	30.77 ± 6.01	yoga (7, 1.5, 6)	30	30.35 ± 5.53	usual care	mFG score, BMI, Hip circumference
Yin MXC, 2021	Hong Kong, China	Rotterdam 2003 consensus criteria	No	9	29.22 ± 2.33	Integrative Body-Mind-Spirit intervention model (1, 3, 6)	9	28.11 ± 3.44	health education information session	PCOSQ domains, Depression (BDI), Anxiety (BAI) BMI, Total testosterone, triglycerides, HDL, LDL, Total cholesterol
Li Y, 2022	China	Rotterdam 2003 consensus criteria	Yes	24	23.2 ± 4.38	tai chi (1, 3, 12)	18	22.9 ± 4.64	Self-monitored exercise	BMI, Weight, Waist-hip ratio, mFG score, LH, FSH, LH/FSH, SHBG, DHEAS, Total testosterone, Fasting blood glucose, Fasting insulin, HOMA-IR, Triglycerides, HDL, LDL, Total cholesterol
Majidzadeh S, 2023	Iran	Unclear	No	42	30.3 ± 5.5	cognitive-behavioral therapy (1, 1-1.5, 8)	42	32.0 ± 4.8	routine treatments	PCOSQ domains, Depression (BDI), Anxiety (STAI-State)

* means data presented as median (25; 75 quartiles). PCOSQ, Polycystic Ovary Syndrome Questionnaire; STAI, State-Trait Anxiety Inventory; BMI, Body Mass Index

mFG score, Modified Ferriman-Gallwey score; FSH, Follicle-Stimulating Hormone; LH, Luteinizing Hormone; HOMA-IR, Homeostatic Model Assessment for Insulin Resistance; HDL, High-Density Lipoprotein; LDL, Low-Density Lipoprotein; DASS 21, Depression, Anxiety, and Stress Scale – 21; PSS-14, Perceived Stress Scale – 14; CES-D, Center for Epidemiologic Studies Depression Scale; SHBG, Sex Hormone-Binding Globulin; MDI, Major Depression Inventory; BAI, Beck Anxiety Inventory; BDI-II, Beck Depression Inventory-II; DHEAS, Dehydroepiandrosterone sulfate

**Fig. 2:**
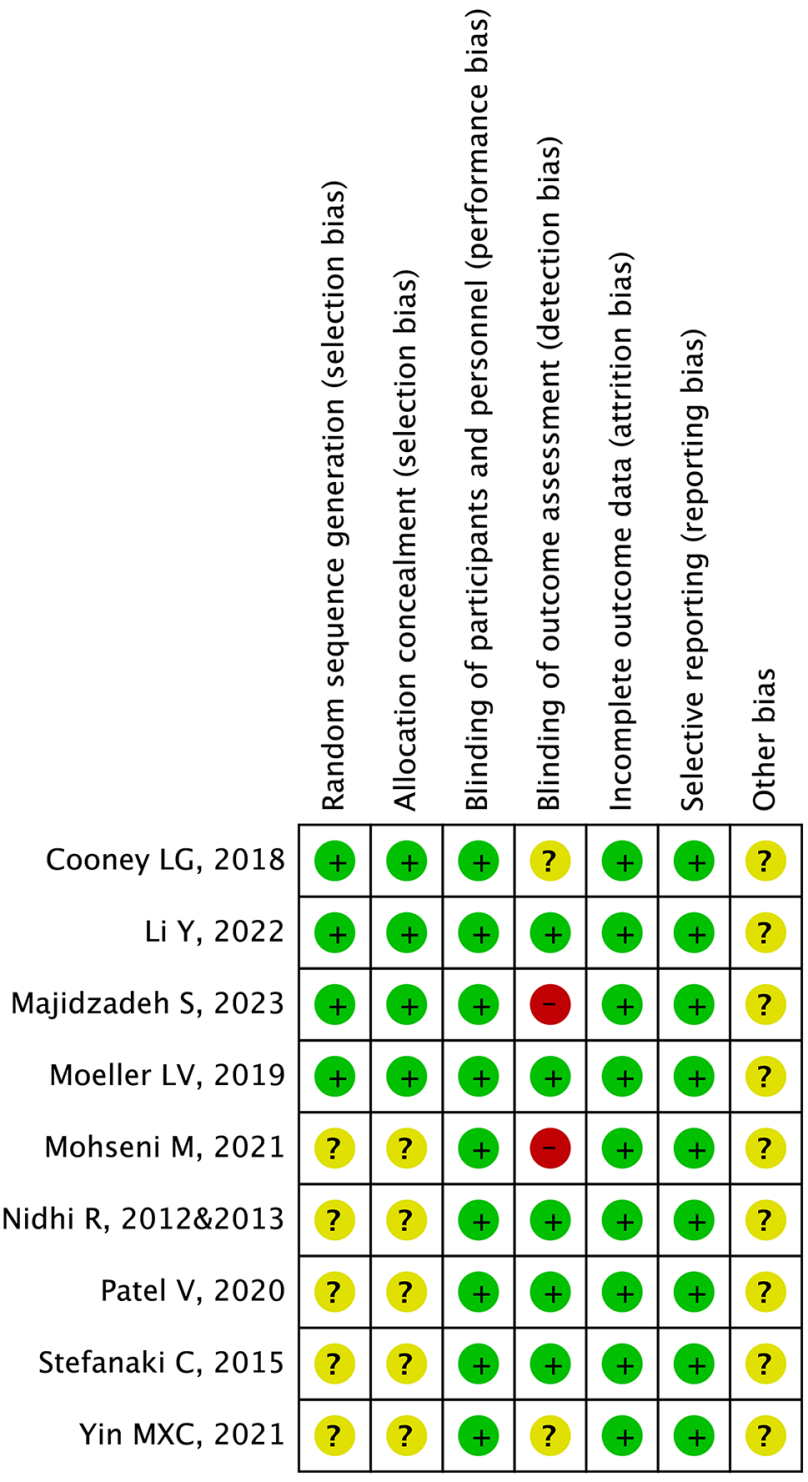
Risk of bias summary of included studies

Regarding the randomization process, four studies did not describe the specific method and only mentioned randomness [36–39, 43–45], leading to some concern classification. Two studies lacked blinding of outcome assessor [21, 44] leading to high risk in D4 and overall results. Additionally, deviations from the intended interventions, data integrity and selective reporting were both assessed as low risk [20, 21, 36–45]. The results are presented in Fig. 2.

### Meta-analysis results

#### Effects of MBI on health-related quality of life of patients with PCOS

**Fig. 3:**
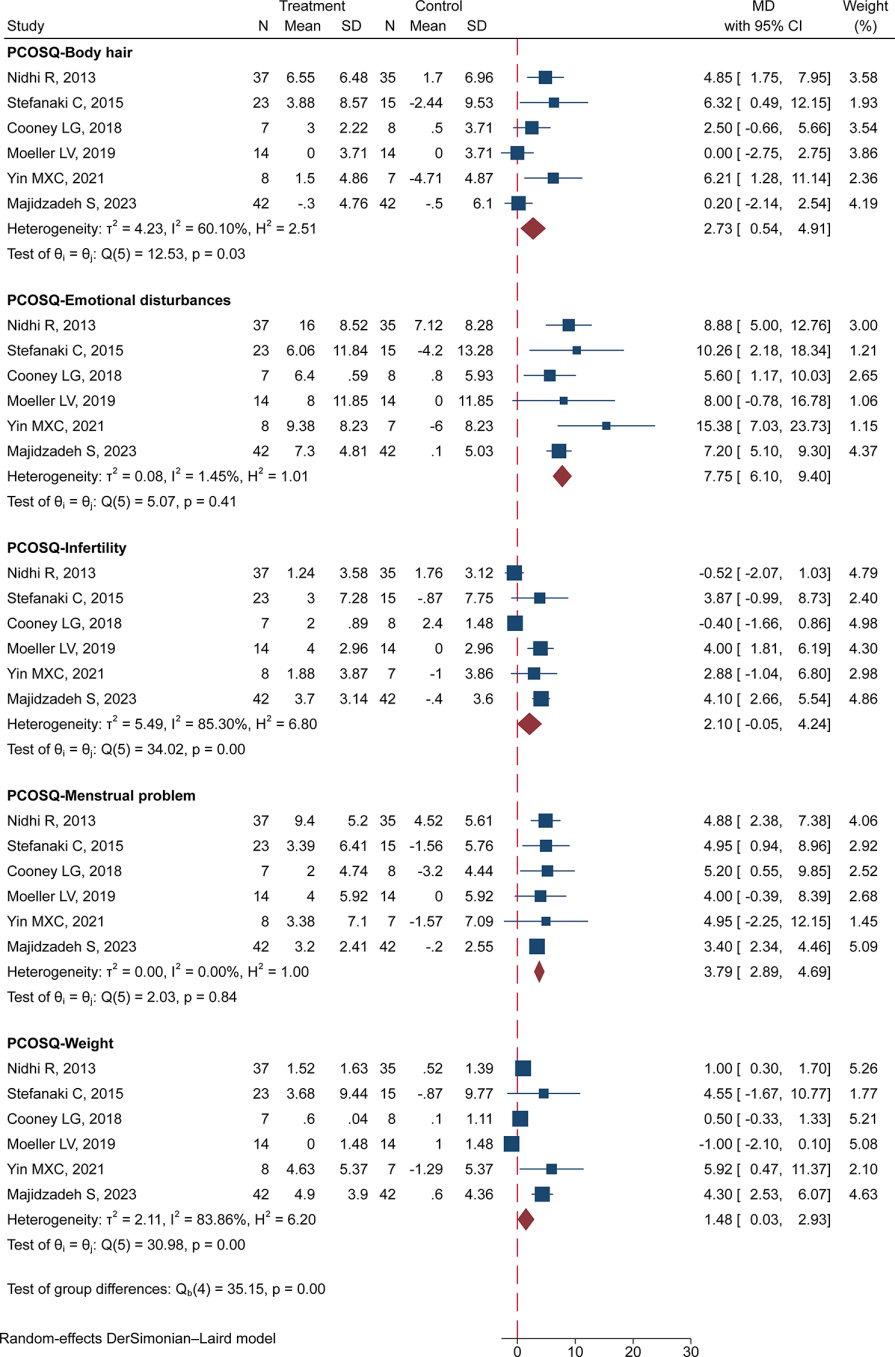
Forest plot of effects of MBI on health-related quality of life of patients with PCOS

It is important to emphasize that we modified the scoring orientation and range of the PCOSQ to facilitate direct comparison using MD in our analysis, where higher scores now represent better function. As a result, our meta-analysis revealed significant enhancements in the health-related quality of life among patients with PCOS following MBI (Fig. 3). These improvements were particularly pronounced in various PCOSQ subscale scores, including emotional disturbances (MD: 7.75, 95% CI: 6.10 to 9.40), body hair (MD: 2.73, 95% CI: 0.54 to 4.91), menstrual problems (MD: 3.79, 95% CI: 2.89 to 4.69), and weight (MD: 1.48, 95% CI: 0.03 to 2.93). Notably, infertility exhibited no significant improvement (MD: 2.10, 95% CI: -0.05 to 4.24).

However, it is crucial to acknowledge that our analysis identified substantial heterogeneity for the outcomes of body hair (I² = 60.1%), infertility (I² = 85.30%), and weight (I² = 83.86%). Conversely, emotional disturbances and menstrual problems exhibited acceptable levels of heterogeneity.

#### Effects of MBI on anxiety, depression, and stress of patients with PCOS

**Fig. 4:**
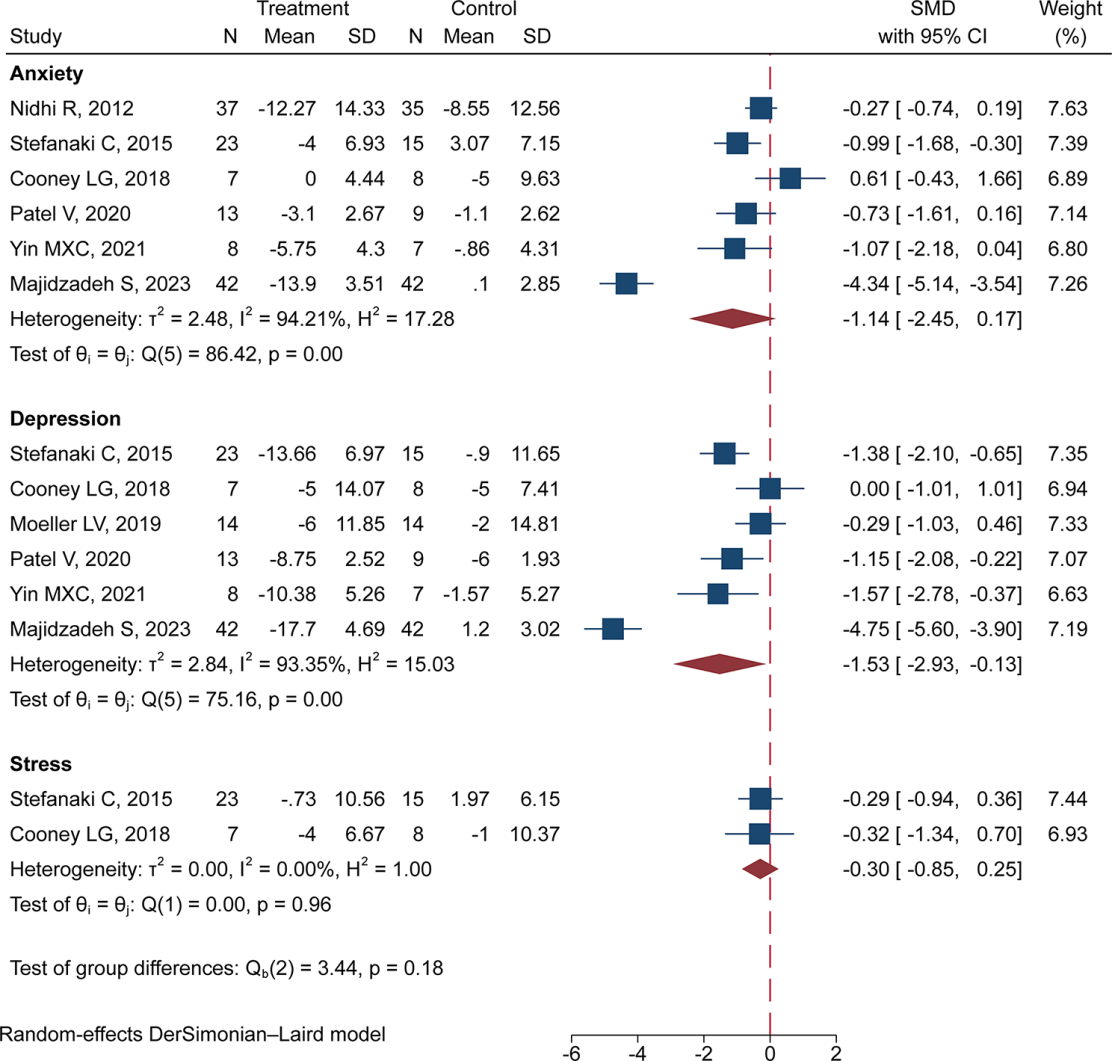
Forest plot of effects of MBI on depression, anxiety, and stress of patients with PCOS

The analysis of MBI on patients with PCOS revealed a statistically significant reduction in depression levels (MD: -1.53, 95% CI: -2.93 to -0.13) with heterogeneity (I^2^ = 93.35%). However, there were no statistically significant changes in anxiety and stress levels (MD: -1.14, 95% CI: -2.45 to 0.17; MD: -0.30, 95% CI: -0.85 to 0.25, respectively). The result is presented in Fig. 4.

#### Secondary outcome results

Table 2 presents the results of the secondary outcome. Among the measured parameters, statistically significant differences were observed in waist-hip ratio (MD: -0.02, 95% CI: -0.03 to -0.01), fasting blood glucose (MD: -0.25, 95% CI: -0.36 to -0.14 mmol/L), and HOMA-IR (MD: -0.69, 95% CI: -1.22 to -0.15) with accepted heterogeneity (I^2^ = 0). However, for endocrine regulation, there were no statistically significant differences as all outcomes did not show significant effects. Furthermore, seven studies reported the results of BMI(MD: -0.24, 95% CI: -0.51 to 0.03). While it was observed that the MBI was able to achieve weight reduction compared to the control group, these differences did not reach statistical significance.

**Table 2:** Secondary outcome results

Outcome categories	Outcome	No. of studies	MD (95%CI)	Heterogeneity (I^2^, %)
Anthropometrics	Weight (kg)	3	0.05 (-0.99, 1.09)	3.1
	BMI	7	-0.24 (-0.51, 0.03)	0
	waist circumference (cm)	2	-0.46 (-1.44, 0.53)	0
	hip circumference (cm)	3	-0.61 (-3.41, 2.19)	43.01
	waist-hip ratio^*^	4	-0.02 (-0.03, -0.01)	0
Endocrine parameters	mFG score	4	-0.78 (-1.61, 0.06)	68.40
	FSH (mIU/mL)	2	-0.23 (-1.27,0.80)	0
	LH	2	-2.68 (-11.56, 6.21)	86.66
	LH/FSH ratio	2	-0.69 (-2.21, 0.83)	66.68
	SHBG (nmol/L)	2	8.87 (-4.99, 22.73)	0
	DHEAS((µg/dL)	2	-16.98 (-51.52, 17.56)	89.58
	Total testosterone(mmol/L)	4	-2.11 (-4.41, 0.18)	91.75
	Free testosterone(pg/mL)	2	-0.94 (-2.30, 0.42)	78.26
glucose metabolism and blood lipid marker	Fasting insulin(µU/ml)	3	-2.88 (-6.31, 0.56)	24.56
	Fasting blood glucose (mmol/L) ^*^	3	-0.25 (-0.36, -0.14)	0
	HOMA-IR^*^	4	-0.69(-1.22, -0.15)	0
	triglycerides(mmol/L) ^*^	4	-0.21 (-0.41, -0.01)	51.96
	HDL (mmol/L)	4	-0.02 (-0.09, 0.05)	0
	LDL (mmol/L)	4	0.04 (-0.27, 0.35)	70.90
	Total cholesterol(mmol/L)	3	0.05 (-0.27, 0.37)	13.26

* means The differences were statistically significant (*P* < 0.05). BMI: Body Mass Index; mFG score: Modified Ferriman-Gallwey score; FSH: Follicle-Stimulating Hormone; LH: Luteinizing Hormone; SHBG: Sex Hormone-Binding Globulin; DHEAS: Dehydroepiandrosterone sulfate; HOMA-IR: Homeostatic Model Assessment of Insulin Resistance; HDL: High-density lipoprotein; LDL: Low-density lipoprotein

### Subgroup analysis and sensitivity analysis

Table 3 depicts the results of subgroup analyses. Due to the limited number of available studies, we conducted subgroup analysis only for the PCOSQ domains, depression, anxiety, and BMI. We did not conduct subgroup analysis for intervention types because there were not at least two intervention types with more than two studies included. The study findings indicate that the weight status of the population itself affects treatment outcomes. In the overweight subgroup, statistical significance for PCOSQ-Body hair, PCOSQ-Weight, and depression changed from being significant in the overall analysis to non-significant (*P* > 0.05). Additionally, a statistically significant difference was observed in BMI reduction among non-overweight women, in contrast to the overall analysis where BMI reduction did not exhibit statistical significance. The risk of bias presented in the included studies may lead to changes in the statistical direction of PCOSQ-Body hair, PCOSQ-Weight, Depression, Anxiety, and BMI. However, due to limitations in the number of studies and potential missing information, further caution is required when interpreting these changes.

**Table 3:** Subgroup analysis of PCOSQ domains, depression, anxiety, and BMI

Subgroup	No. of studies	MD (95% CI)	Heterogeneity (I^2^, %)
**PCOSQ- Body hair**			
Overall	6	2.73 (0.54, 4.91)	60.10
**Overweight women**			
Yes	2	1.12 (-1.31, 3.56)	27.03
No	4	3.89 (0.54, 7.25)	68.92
**Omit high risk**			
Yes	4	1.36 (-0.69, 3.40)	40.26
No	2	5.34 (2.61, 7.86)	0
**PCOSQ- Emotional disturbances**			
Overall	6	7.75 (6.10, 9.40)	1.45
**Overweight women**			
Yes	2	6.09 (2.13, 10.04)	0
No	4	8.61 (6.10, 11,12)	25.94
**Omit high risk**			
Yes	4	7.12 (5.31, 8.93)	0
No	2	11.04 (5.04, 17.03)	47.76
**PCOSQ- Infertility**			
Overall	6	2.10 (-0.05, 4.24)	85.30
**Overweight women**			
Yes	2	1.70 (-2.60, 6.01)	91.40
No	4	2.40 (-0.59, 5.38)	84.15
**High risk studies**			
No	4	2.73 (-0.12, 5.58)	88.37
Yes	2	0.68 (-2.50, 3.87)	59.94
**PCOSQ-Menstrual problems**			
Overall	6	3.79 (2.89, 4.69)	0
**Overweight women**			
Yes	2	4.57 (1.38, 7,75)	0
No	4	3.72 (2.78, 4.66)	0
**High risk studies**			
No	4	3.60 (2.62, 4.58)	0
Yes	2	4.89 (2.53, 7.25)	0
**PCOSQ-Weight**			
Overall	6	1.48 (0.03, 2.93)	83.86
**Overweight women**			
Yes	2	-0.20 (-1.67, 1.26)	78.19
No	4	3.31 (0.62, 6.00)	80.01
**High risk studies**			
No	4	1.47 (-0.79, 3.74)	88.69
Yes	2	2.69 (-1.89, 7.27)	67.56
**Depression**			
**Subgroup**	**No. of studies**	**SMD (95% CI)**	**Heterogeneity (I**^**2**^, **%)**
Overall	6	1.53 (-2.93, -0.13)	93.35
**Overweight women**			
Yes	3	-0.48 (-1.12, 0.16)	35.56
No	3	-2.58 (-4.86, -0.29)	94.75
**High risk studies**			
No	4	-1.61( -3.65, 0.44)	95.97
Yes	2	-1.31 (-2.04, -0.57)	0
**Anxiety**			
**Subgroup**	**No. of studies**	**SMD (95% CI)**	**Heterogeneity (I**^**2**^, **%)**
Overall	6	1.14 (-2.45, 0.17)	94.21
**Overweight women**			
Yes	2	-0.09 (-1.40, 1.22)	72.79
No	4	-1.66 (-3.45, 0.13)	96.02
**High risk studies**			
No	3	-1.58 (-4.31, 1.15)	96.91
Yes	3	-0.47 (-0.89, -0.06)	6.35
**BMI**			
**Subgroup**	**No. of studies**	**MD (95% CI)**	**Heterogeneity (I**^**2**^, **%)**
Overall	7	-0.24 (-0.51, 0.03)	0
**Overweight women**			
Yes	4	0.22 (-0.30, 0.75)	0
No	3	-0.40 (-0.70, -0.09)	0
**High risk studies**			
No	3	0.27 (-0.27, 0.81)	0
Yes	4	-0.40(-0.70, -0.09)	0

**Fig. 5:**
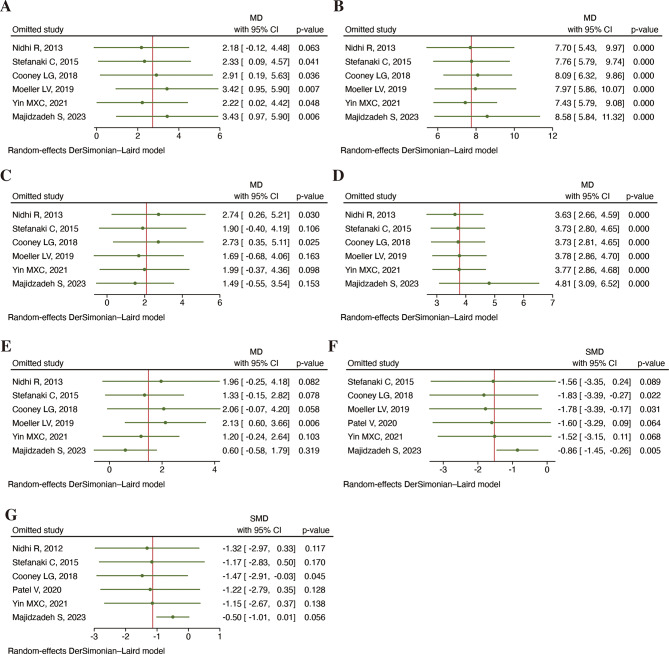
Sensitivity analysis

The sensitivity analysis conducted through a stepwise exclusion method, revealed the robustness of the results for PCOSQ-Emotional disturbances and PCOSQ-Menstrual problems (*P* < 0.001, Fig. 5).

### GRADE results

The GRADE level of evidence is high for PCOSQ-Emotional disturbances, and PCOSQ-Menstrual problems, while it is moderate for anxiety. Notably, the level of evidence for other primary outcome measures is generally low. Table S2 shows the GRADE evidence profiles.

### Publication bias

We did not perform a publication bias assessment because all included measures had fewer than 10 studies.

## Discussion

To our knowledge, this study represents the first systematic evaluation of the therapeutic benefits of MBTs on body image, androgen secretion, glucose and lipid metabolism, and quality of life in patients with PCOS. This meta-analysis consisted mainly of 12 articles involving 311 patients. Substantial and significant improvements were observed in various aspects of health-related quality of life, especially in emotional disturbances, body hair, menstrual problems, and weight. Notably, the improvements in emotional disturbances and menstrual problems were robust and received a high grade of evidence. Statistically significant differences were observed in reducing depression, waist-hip ratio, fasting blood glucose, and HOMA-IR. However, there were no significant differences in endocrine hormone secretion. Subgroup analysis revealed that being overweight significantly impacted the outcomes, leading to a loss of statistical differences in PCOSQ-Body hair and PCOSQ-Weight and affecting BMI reduction.

Patients with PCOS often experience a diminished quality of life and are at an increased risk of mental health issues due to physical and emotional challenges. Mind-body approaches represent a promising and readily embraced alternative to traditional mental health interventions. Multiple guidelines and reviews have consistently demonstrated the effectiveness of mind-body approaches in alleviating symptoms of depression, anxiety, and various chronic health issues [17, 46, 47]. Previous research has reported that cognitive behavioral therapy can improve depression in PCOS [48], which is consistent with our study’s findings. Our results also indicate that MBI can potentially alleviate emotional distress and menstrual issues, thereby enhancing the overall quality of life – a fact that has received limited attention in previous meta-analyses. Extensive research has consistently demonstrated that regardless of the specific PCOS subtype, there exists a close association with insulin resistance, type 2 diabetes, and cardiovascular diseases [3, 49]. Insulin resistance is a condition in which the body does not respond effectively to insulin, leading to elevated insulin levels in the bloodstream and in turn, can result in various metabolic consequences, including difficulties in regulating blood sugar levels. Among the parameters we investigated, our research suggests that MBI can have a positive impact on improving fasting blood glucose levels and HOMA-IR. However, no significant effect on fasting insulin levels was observed. This implies that MBI may potentially lower blood glucose levels by enhancing insulin sensitivity. Anita Verma’s study also reported a potentially significant decrease in menstrual irregularity, clinical hyperandrogenism, fasting blood glucose, fasting insulin, and HOMA-IR values [19]. While our findings did not align with their results regarding fasting insulin levels, a meticulous data verification process revealed that their analysis focused solely on endpoint data, overlooking potential statistical differences arising from variations in individual baseline data. Upon reanalysis, we found that the differences in fasting insulin did not hold statistical significance, aligning with our results. However, it’s crucial to acknowledge that since this is a meta-analysis of RCT, it does not conclusively demonstrate that MBI has no effect on fasting insulin level. Furthermore, our study primarily included practices such as yoga, tai chi, and cognitive-behavioral therapy. Thus, there is a need for further research to explore the effects of other mind-body interventions. Additionally, due to the limited number of studies in our analysis, the mechanisms underlying the reduction in blood glucose levels require further exploration. Women with PCOS who had higher BMI scores experienced lower body satisfaction and improve body image can effectively regulate their mental health [50]. Our research indicates that while MBI does not exhibit a statistically significant reduction in BMI within the overall study population, it does lead to a reduction in BMI among non-overweight individuals. Additionally, MBI has been found to lower the waist-hip ratio. It indicates that MBI could be a valuable approach for individuals who are not overweight but may still seek improvements in body composition and metabolic health. Furthermore, the observed reduction in the waist-hip ratio indicates a potential positive effect of MBI on body fat distribution, which can causes additional disorders in metabolic and hormonal parameters in PCOS women [51]. Obesity is a significant factor contributing to an elevated risk of reduced fertility and infertility, with obese women consistently experiencing poorer reproductive outcomes, regardless of the method of conception [52]. Our research has further enriched this discovery, as within the overweight subgroup, various parameters such as PCOSQ-Body hair, PCOSQ-Weight, and depression were adversely affected. For PCOS patients, the management of weight can be considered a crucial component of their overall care. Implementing weight control methods alongside interventions like MBI therapy may offer a comprehensive approach to addressing the complex challenges associated with PCOS. Future research should continue to explore the synergistic effects of these strategies and their potential to optimize the well-being and treatment response of PCOS patients. Endocrine hormone levels in the body are influenced by various factors, including diet, exercise, medication, sleep, mental stress, and fatigue [53]. Our study did not observe any significant effects on mFG score, FSH, LH, LH/FSH ratio, SHBG, or DHEAS. This could potentially be attributed to the limited availability of consolidated data, individual variability and differences, and potential time-dependent effects. Future research should focus on more precise and in-depth investigations to better understand the potential role of mind-body therapy in PCOS in relation to endocrine parameters.

In forthcoming trials, it is imperative to consider various intervention characteristics, including the influence of intervention type, duration, intensity, and other implementation aspects on various outcomes. While our meta-analysis primarily compared MBI with placebos or routine care, future research should encompass more comparisons of similar types of MBI with consistent control interventions. Extrapolating these results to standard drug therapies can hold significant clinical implications. Furthermore, more attention should be dedicated to the study of ovulation, menstrual patterns, and reproductive outcomes. Additionally, including cost-benefit analyses in the results and comparing them with commonly used drugs and surgical treatments can provide valuable insights into the efficacy and economic considerations associated with PCOS management.

Our study provided a comprehensive summary of the evidence regarding the use of MBI in the treatment of PCOS. However, it is crucial to acknowledge several noteworthy limitations in our study. Firstly, our meta-analysis incorporated a limited number of studies characterized by small sample sizes. In many instances, the effect was assessed by very few studies; thus, the evidence to support it is low. Additionally, significant heterogeneity was encountered, perhaps due to various regimens, doses, durations, center settings, populations enrolled, etc. These factors can potentially compromise the robustness of our findings and significantly undermine the validity of the results. Secondly, our study only included three yoga studies and two cognitive-behavioral therapy studies, along with other therapies with one study. This limited diversity in intervention methods hampers our ability to conduct more nuanced subgroup analyses based on intervention type and leaves a lack of evidence to explore the therapeutic effects of other mind-body interventions. Thirdly, inadequate reporting of bias risk in the included studies is a potential concern and many of the studies suffer from significant sources of bias that may introduce bias into the results. Future research should prioritize adherence to CONSORT standards for the comprehensive reporting of randomized controlled trials [54].

## Conclusion

This meta-analysis suggests that MBI represents a promising alternative therapy for patients with PCOS. MBI shows beneficial effects on the quality of life and depression in PCOS patients, as well as reductions in waist-hip ratio, fasting blood glucose, and HOMA-IR. However, future well-designed, high-quality, large-scale studies are needed to comprehensively assess the impact of different MBIs on various PCOS phenotypes.

### Electronic supplementary material

Below is the link to the electronic supplementary material.

Supplementary Material

## Data Availability

No datasets were generated or analysed during the current study.

## Abbreviations

PCOS: Polycystic ovary syndrome
TG: Triglycerides
LDL: Low-density lipoprotein cholesterol
HDL: High-density lipoprotein
MBI: Mind-body interventions
CBT: Cognitive-behavioral therapy
PRISMA: PREFERRED Reporting Items for Systematic Reviews and Meta-analyses
MeSH: Medical Subject Headings
PCOSQ: Polycystic Ovary Syndrome Questionnaire
BMI: Body Mass Index
mFG: Modified Ferriman-Gallwey
FSH: Follicle- Stimulating Hormone
LH: Luteinizing Hormone
SHBG: Sex Hormone-Binding Globulin
DHEAS: Dehydroepiandrosterone Sulfate
HOMA-IR: Homeostatic Model Assessment for Insulin Resistance
RCT: Randomized controlled trial
ROB: Risk of bias
GRADE: Grading of Recommendations Assessment, Development and Evaluation
SD: Standard deviation
MD: Mean differences
CI: Confidence Intervals
SMD: Standardized mean differences
I-BMS: Integrative Body-Mind-Spirit
