# Supplementary material for: Effects of mind-body interventions on polycystic ovary syndrome: a comprehensive meta-analysis
Source: J Ovarian Res. 2024 Jul 25;17:154. doi: 10.1186/s13048-024-01477-2 (PMC11271059; doi:10.1186/s13048-024-01477-2)
Supplement: Supplementary file 1 — Supplementary Material [file 13048_2024_1477_MOESM1_ESM.docx]

**Table S1. Search strategy**

| **Database** | **#** | **Search strategy** | **Results** |
| --- | --- | --- | --- |
| PubMed | 1 | "Polycystic Ovary Syndrome"[MeSH Terms] | 18964 |
|  | 2 | "polycystic ovar*"[Title/Abstract] OR "PCOS"[Title/Abstract] OR "PCOD"[Title/Abstract] OR ("sclerocystic"[Title/Abstract] AND "ovar*"[Title/Abstract]) OR "stein Leventhal"[Title/Abstract] | 25111 |
|  | 3 | #1 OR #2 | 26670 |
|  | 4 | "Mind-Body Therapies"[MeSH Terms] OR "Yoga"[MeSH Terms] OR "biofeedback, psychology"[MeSH Terms] OR "Counseling"[MeSH Terms] OR "Aromatherapy"[MeSH Terms] OR "Tai Ji"[MeSH Terms] OR "Cognitive Behavioral Therapy"[MeSH Terms] OR "Psychotherapy"[MeSH Terms] OR "Relaxation"[MeSH Terms] OR "Relaxation Therapy"[MeSH Terms] | 300443 |
|  | 5 | ("mind"[Title/Abstract] AND "body"[Title/Abstract]) OR "hypnosis"[Title/Abstract] OR "meditat*"[Title/Abstract] OR "relax*"[Title/Abstract] OR "mindful*"[Title/Abstract] OR "yoga"[Title/Abstract] OR "tai chi"[Title/Abstract] OR ("breath*"[Title/Abstract] AND "exercise*"[Title/Abstract]) OR "massage*"[Title/Abstract] OR "imagery"[Title/Abstract] OR "biofeedback"[Title/Abstract] OR "hypno*"[Title/Abstract] OR "suggest*"[Title/Abstract] OR "autosuggest*"[Title/Abstract] OR "aromatherapy"[Title/Abstract] | 5221598 |
|  | 6 | #4 OR #5 | 5445455 |
|  | 7 | (randomized controlled trial[Publication Type] OR controlled clinical trial[Publication Type] OR randomized[Title/Abstract] OR placebo[Title/Abstract] OR clinical trials as topic[Mesh:NoExp] OR randomly[Title/Abstract] OR trial[Title]) NOT (animals [Mesh] NOT (humans[Mesh] AND animals[Mesh])) | 1497645 |
|  | 8 | #3 AND #6 AND #7 | 522 |
|  | 1 | MeSH descriptor: [Polycystic Ovary Syndrome] explode all trees | 2157 |
| Cochrane library | 2 | (polycystic ovar*):ti,ab,kw OR (PCOS):ti,ab,kw OR (PCOD):ti,ab,kw OR (sclerocystic AND ovar*):ti,ab,kw OR ("stein Leventhal"):ti,ab,kw | 5730 |
|  | 3 | #1 OR #2 | 5730 |
|  | 4 | MeSH descriptor: [Mind-Body Therapies] explode all trees | 9453 |
|  | 5 | MeSH descriptor: [Yoga] explode all trees | 1230 |
|  | 6 | MeSH descriptor: [Biofeedback, Psychology] explode all trees | 2178 |
|  | 7 | MeSH descriptor: [Counseling] explode all trees | 7589 |
|  | 8 | MeSH descriptor: [Aromatherapy] explode all trees | 402 |
|  | 9 | MeSH descriptor: [Tai Ji] explode all trees | 585 |
|  | 10 | MeSH descriptor: [Cognitive Behavioral Therapy] explode all trees | 14178 |
|  | 11 | MeSH descriptor: [Psychotherapy] explode all trees | 35739 |
|  | 12 | MeSH descriptor: [Relaxation] explode all trees | 2195 |
|  | 13 | MeSH descriptor: [Relaxation Therapy] explode all trees | 2705 |
|  | 14 | #4 OR #5 OR #6 OR #7 OR #8 OR #9 OR #10 OR #11 OR #12 OR #13 | 46635 |
|  | 15 | (mind AND body):ti,ab,kw OR hypnosis:ti,ab,kw OR meditat*:ti,ab,kw OR relax*:ti,ab,kw OR mindful*:ti,ab,kw OR yoga:ti,ab,kw OR tai chi:ti,ab,kw OR (breath* AND exercise*):ti,ab,kw OR massage*:ti,ab,kw OR imagery:ti,ab,kw OR biofeedback:ti,ab,kw OR hypno*:ti,ab,kw OR suggest*:ti,ab,kw OR autosuggest*:ti,ab,kw OR aromatherapy:ti,ab,kw | 287500 |
|  | 16 | #14 OR #15 | 315018 |
|  | 17 | #3 AND #16 | 664 |
| EMBASE | 1 | 'ovary polycystic disease'/exp | 40,069 |
|  | 2 | 'polycystic ovar*':ab,ti OR 'pcos':ab,ti OR 'pcod':ab,ti OR (sclerocystic:ab,ti AND ovar*:ab,ti) OR 'stein leventhal':ab,ti | 36,590 |
|  | 3 | #1 OR #2 | 45,535 |
|  | 4 | 'autogenic training'/exp OR 'guided imagery'/exp OR 'relaxation training'/exp OR 'imagery'/exp OR 'suggestion'/exp OR 'hypnosis'/exp OR 'meditation'/exp OR 'tai chi'/exp OR 'counseling'/exp OR 'awareness'/exp OR 'bodywork'/exp OR 'massage'/exp | 429,030 |
|  | 5 | mind:ab,ti AND body:ab,ti OR hypnosis:ab,ti OR meditat*:ab,ti OR relax*:ab,ti OR mindful*:ab,ti OR yoga:ab,ti OR 'tai chi':ab,ti OR (breath*:ab,ti AND exercise*:ab,ti) OR massage*:ab,ti OR imagery:ab,ti OR biofeedback:ab,ti OR hypno*:ab,ti OR suggest*:ab,ti OR autosuggest*:ab,ti OR aromatherapy:ab,ti | 6,499,445 |
|  | 6 | #4 OR #5 | 6,825,855 |
|  | 7 | 'crossover procedure':de OR 'double-blind procedure':de OR 'randomized controlled trial':de OR 'single-blind procedure':de OR random*:de,ab,ti OR factorial*:de,ab,ti OR crossover*:de,ab,ti OR ((cross NEXT/1 over*):de,ab,ti) OR placebo*:de,ab,ti OR ((doubl* NEAR/1 blind*):de,ab,ti) OR ((singl* NEAR/1 blind*):de,ab,ti) OR assign*:de,ab,ti OR allocat*:de,ab,ti OR volunteer*:de,ab,ti | 3,363,039 |
|  | 8 | #3 AND #6 AND #7 | 1,436 |

**Search date: July 6, 2024**

**Table S2. The result of GRADE assessment.**

| **Outcome** | **Certainty assessment** | | | | | | | **№ of patients** | | **Effect** | | **Certainty** |
| --- | --- | --- | --- | --- | --- | --- | --- | --- | --- | --- | --- | --- |
|  | **№ of studies** | **Study design** | **Risk of bias** | **Inconsistency** | **Indirectness** | **Imprecision** | **Other considerations** | **MBI** | **Control** | **Relative**  **(95% CI)** | **Absolute**  **(95% CI)** |  |
| **PCOSQ-body hair** | 6 | randomized trials | not serious | serious^a^ | not serious | serious^b^ | none | 131 | 121 | - | MD **2.73**  (0.54 to 4.91) | ⨁⨁◯◯  Low |
| **PCOSQ-Emotional disturbances** | 6 | randomized trials | not serious | not serious | not serious | not serious | none | 131 | 121 | - | MD **7.75**  (6.1 to 9.4) | ⨁⨁⨁⨁  High |
| **PCOSQ-Infertility** | 6 | randomized trials | not serious | serious^a^ | not serious | serious^b^ | none | 131 | 121 | - | MD **2.1**  (-0.05 to 4.24) | ⨁⨁◯◯  Low |
| **PCOSQ-Menstrual problems** | 6 | randomized trials | not serious | not serious | not serious | not serious | none | 131 | 121 | - | MD **3.79**  (2.89 to 4.69) | ⨁⨁⨁⨁  High |
| **PCOSQ-Weight** | 6 | randomized trials | not serious | serious^a^ | not serious | serious^b^ | none | 131 | 121 | - | MD **1.48**  (0.03 to 2.93) | ⨁⨁◯◯  Low |
| **Depression** | 6 | randomized trials | not serious | serious^a^ | not serious | serious^b^ | none | 107 | 95 | - | SMD **1.53**  (-2.93 to -0.13) | ⨁⨁◯◯  Low |
| **Anxiety** | 6 | randomized trials | not serious | serious^a^ | not serious | not serious | none | 130 | 116 | - | SMD **1.14**  (-2.45 to 0.17) | ⨁⨁⨁◯  Moderate |

**CI:** confidence interval; **MD:** mean difference; **SMD:** standardized mean difference. a. a high level of heterogeneity; b. the confidence interval (CI) in a GRADE assessment is very close to the null effect line
